# Autophagic Killing Effects against *Mycobacterium tuberculosis* by Alveolar Macrophages from Young and Aged Rhesus Macaques

**DOI:** 10.1371/journal.pone.0066985

**Published:** 2013-06-18

**Authors:** Sophia A. Pacheco, Katelyn M. Powers, Flora Engelmann, Ilhem Messaoudi, Georgiana E. Purdy

**Affiliations:** 1 Department of Molecular Microbiology and Immunology, Oregon Health and Sciences University, Portland, Oregon, United States of America; 2 Division of Biomedical Sciences, University of California Riverside, Riverside, California, United States of America; 3 Oregon National Primate Research Center, Division of Pathobiology and Immunology, Beaverton, Oregon, United States of America; Bose Institute, India

## Abstract

Non-human primates, notably rhesus macaques (*Macaca mulatta*, RM), provide a robust experimental model to investigate the immune response to and effective control of *Mycobacterium tuberculosis* infections. Changes in the function of immune cells and immunosenescence may contribute to the increased susceptibility of the elderly to tuberculosis. The goal of this study was to examine the impact of age on *M. tuberculosis* host-pathogen interactions following infection of primary alveolar macrophages derived from young and aged rhesus macaques. Of specific interest to us was whether the mycobactericidal capacity of autophagic macrophages was reduced in older animals since decreased autophagosome formation and autophagolysosomal fusion has been observed in other cells types of aged animals. Our data demonstrate that alveolar macrophages from old RM are as competent as those from young animals for autophagic clearance of *M. tuberculosis* infection and controlling mycobacterial replication. While our data do not reveal significant differences between alveolar macrophage responses to *M. tuberculosis* by young and old animals, these studies are the first to functionally characterize autophagic clearance of *M. tuberculosis* by alveolar macrophages from RM.

## Introduction

*Mycobacterium tuberculosis* is a highly successful pathogen that infects one-third of the world's population. The majority of individuals that are exposed to *M. tuberculosis* mount a successful immune response that contains the bacterium in a latent state. Reactivation or progression occurs when the host immune response is impaired, for example upon co-infection with HIV or following changes in the immune system that are associated with aging. A hallmark of *M. tuberculosis* pathogenesis is its ability to infect and survive within the host macrophage by preventing fusion of the *M. tuberculosis*-containing vacuole with the lysosome. Instead *M. tuberculosis* resides in a vacuole that resembles an early endosome with a pH = 6.4 and retains markers such as the Rab5 GTPase [1]–[4]. While the pathogen is well-adapted to modulate host vesicular trafficking in resting macrophages, immune activation shifts the balance towards mycobacterial clearance [5], [6]. Activated mouse macrophages promote killing of mycobacteria via the production of reactive oxygen and nitrogen intermediates, and by delivering the bacterium to the lysosome. IFN-γ activation also induces the cellular process macroautophagy (hereafter referred to as autophagy), a mechanism by which the eukaryotic cell degrades damaged proteins and organelles by delivering them in a vacuole called the autophagosome to the lysosome. Autophagy contributes to innate immunity by controlling infections by some viruses, intracellular bacteria and parasites [7]. The induction of autophagy in *M. bovis* BCG- and *M. tuberculosis*-infected macrophages by serum-starvation, rapamycin-treatment, or IFN-γ activation leads to killing of intracellular bacteria as a result of trafficking bacteria to the *lysosome* [8], [9]. The lysosome is a highly toxic environment characterized by low pH, and enriched in hydrolytic enzymes. In addition, we and others recently demonstrated the mycobactericidal contribution of antimicrobial peptides generated in the lysosome [8], [10].

While the mouse model of infection has contributed greatly to our understanding of mycobacterial pathogenesis and immune control of tuberculosis infection, extensive literature documents differences in immune control of the infection between humans and rodents. At the level of *M. tuberculosis*-macrophage interactions, there are likely significant differences between the mycobactericidal repertoire found in mice and humans. Reactive nitrogen intermediates (RNI) are produced by activated murine macrophages and contribute to bacterial clearance. In human infections, RNI are present in the granuloma, but it has been difficult to establish a presence or a role for RNI within human macrophages [11]. Mycobactericidal compounds present in humans and not in mice include the antimicrobial peptides granulysin, human neutrophil peptides (HNP1–3) and the cathelicidins [12]. Finally, *M. tuberculosis* infections of mice are clinically different from human tuberculosis resulting in a chronic persistent infection, whereas *M. tuberculosis* infection of humans is often latent.

On the other hand, aerosol infection of non-human primates (NHP) results in a latent infection that closely resembles the natural infection of humans. Histopathological analysis of granulomatous lesions in NHP revealed hypoxic, caseous granulomas that are similar to lesions described for human disease [13]. Consequently, there is renewed interest in using NHP models to study the host immune response to *M. tuberculosis* infection and test vaccine and drug therapies [14], [15]. In addition to their wide use for testing potential vaccine candidates or therapeutics, macaques are increasingly used to investigate the underlying immunology of TB/AIDS co-infection and progression of tuberculosis infection [16]–[19]. It is likely that NHP will also prove valuable in defining and examining determinants of mycobacterial pathogenesis. Dutta et al. recently performed a small-scale transposon mutant screen to identify *M. tuberculosis* virulence factors [20]. They identified a greater number of *M. tuberculosis* mutants as attenuated in the macaque compared to those identified in previous studies using mice. Therefore, use of the NHP model will not only provide a more nuanced interpretation of the host immune response, but will likely elucidate novel *M. tuberculosis* pathways and effectors required for establishment and maintenance of infection.

The primary goal of this study was to examine *M. tuberculosis* host-pathogen interactions in the context of alveolar macrophages from Rhesus macaques (RM). It is anticipated that using relevant tissue macrophages from a closely related species will provide valuable insight into human tuberculosis infection. Specifically, we determined that autophagic clearance of *M. tuberculosis* occurs in alveolar macrophages from RM. These results recapitulate our previous experiments performed using murine bone marrow-derived macrophages [8], and suggest autophagic clearance by macrophages is conserved between mice and humans. Having established a RM alveolar macrophage model, we used it to ask whether primary alveolar macrophages from aged animals were as effective at controlling *M. tuberculosis* infection as those from young animals. Of particular interest was whether the mycobactericidal capacity of autophagic macrophages was reduced in older animals. Age-associated decline of the immune system, or immunosenescence, primarily affects the adaptive immune system, but there are also documented changes in innate immune cell function [21]. With regards to autophagy, decreased autophagosome formation and reduced fusion of autophagosomes with the lysosome has been observed in cells from aged animals. In addition, the lysosomes of aged cells have a reduced concentration of hydrolytic enzymes, which may reduce the bactericidal capacity of this compartment [22], [23]. In contrast to these previous studies, results presented herein indicate that there are not significant differences between alveolar macrophage responses to *M. tuberculosis* by young and old animals.

## Materials and Methods

### Ethics Statement

Experiments involving *M. tuberculosis* were performed in the BSL3 facility at Oregon Health and Science University and approved by the Institutional Biosafety Committee. Non-human primates (NHPs) at the Oregon National Primate Research Center (ONPRC) are handled in strict accordance with the recommendations of the National Institutes of Health's (NIH) “Guide for the Care and use of Laboratory Animals” and the U.S. Animal Welfare Act. The ONPRC is an American Association for Accreditation of Laboratory Animal Care (AAALAC)-accredited, NIH-supported NHP research facility. Bronchial alveolar lavages were performed post-euthanasia on NHPs scheduled for regular necropsy. Euthanasia was performed by trained ONPRC veterinarians using a sodium pentobarbital overdose (greater than 50 mg/kg body weight) followed by exsanguination. This method is consistent with guidelines established by the Panel on Euthanasia of the American Veterinary Medical Association and is approved by the Institutional Animal Care and Use Committee at the ONPRC. Euthanasia was not performed by any of the authors of this manuscript, and none of the animals were assigned to the principal investigator's study.

### Maintenance of bacterial cultures and cells

*M. tuberculosis* wild type strain CDC1551 was maintained in Middlebrook 7H9 liquid medium (Difco) or on Middlebrook 7H10 agar (Difco) plates supplemented with OADC (BD).

### Alveolar macrophage isolation

Alveolar macrophages isolated from rhesus macaques were routinely cultured in RPMI-1640 supplemented with 10% heat-inactivated fetal calf serum, 1% L-glutamine, 0.2% penicillin, 0.05% gentamicin, and 0.2% amphotericin B. Macrophages constituted between 75–90% of bronchial alveolar lavage (BAL) cells by flow cytometric analysis using a gate on size and/or CD14+ and HLA-DR+ immunostaining. We isolated alveolar macrophages from cryopreserved BAL samples by allowing them to adhere to Primaria (Becton Dickinson) tissue culture dishes. Non-adherent cells were removed and the remaining adherent cells were confirmed as >99% alveolar macrophages by flow cytometry as CD14+ and HLA-DR+. To perform immunofluorescence microscopy, alveolar macrophages were adhered onto chamberslides (ibidi). Aged non-human primates (NHP) were defined as >16 years old; young NHP were defined as 2–9 years old. The age and sex of NHP used in this study are summarized in Table S1.

### Macrophage infection conditions

For autophagy studies, alveolar macrophages were infected in triplicate at the indicated MOI (1∶1, 5∶1, or 10∶1). After 1 h incubation to allow for phagocytosis, cells were washed three times with phosphate buffered saline (PBS) to remove extracellular bacteria and then incubated at 37°C in standard culture medium lacking antibiotics (Control) or standard medium containing 50 µg/mL rapamycin (Rap, Santa Cruz) to induce autophagy. Where indicated, the autophagy inhibitors 3-methyl adenine (3-MA, Sigma) and Bafilomycin A (Baf, LC Laboratories) were added at 10 mM and 0.1 µM final concentration, respectively. Stock solutions were made in DMSO at concentrations such that the final concentration of DMSO was less than 0.01%. These conditions did not impair alveolar macrophage viability in the presence or absence of *M. tuberculosis* infection as assessed using cytotoxicity assays or light microscopy in the presence of trypan blue. We visually assessed the health of infected macrophages over the course of the experiment and did not observe loss of viability in terms of detached macrophages. The number of viable bacteria after 4 h treatment was determined by harvesting infected monolayers with PBS containing 0.1% Tween 80 and plating serial dilutions on 7H10 agar (Difco) containing ADS (0.5% BSA, 0.2% dextrose, 0.085% NaCl) supplements. Survival of *M. tuberculosis* in autophagic macrophages was expressed relative to survival of *M. tuberculosis* in macrophages maintained in standard macrophage medium lacking antibiotics (control macrophages).

For infection of alveolar macrophages with *M. tuberculosis*, cells were seeded in 96-well plates and infected in triplicate at an MOI of 5∶1, then incubated at 37°C in standard culture medium. We visually assessed the infected macrophages over the course of the experiment and did not observe loss of viability in terms of detached macrophages. The number of viable bacteria at the indicated timepoint was determined by harvesting infected monolayers with PBS containing 0.1% Tween 80 and plating serial dilutions on 7H10 agar containing ADS supplements.

### Western blot analysis

To quantify autophagy levels in resting (steady-state) and autophagic macrophages, Western blotting of cell lysates was performed. Cell lysates from 2×10^5^ resting or autophagic alveolar macrophages were resolved by SDS-15% PAGE, transferred to nitrocellulose, and probed with rabbit polyclonal antibody against LC3 (Sigma, L7543), or with rabbit polyclonal antibody against actin (Sigma, A5060). HRP-conjugated anti-rabbit secondary antibody was used at a 1∶10,000 dilution. Densitometry was performed using the ImageJ software package (NIH).

### Immunofluorescence microscopy

Immunofluorescence microscopy was performed on resting and autophagic alveolar macrophages from young and old RM. For immunofluorescence studies macrophages were infected with *M. tuberculosis* that was stained beforehand with CMFDA (Molecular Probes). Infected alveolar macrophages in chamberslides (ibidi) were fixed with 4% paraformaldehyde, permeabilized with 5 mM N-octyl glucopyranoside (Sigma), and blocked with PBS containing 0.5% gelatin. Mouse monoclonal antibody against human LAMP-1 (Santa Cruz, H5G11) and rabbit polyclonal antibody against LC3 (Sigma, L7543) were used at a 1∶200 dilution. DyeLight 594-conjugated goat anti-rabbit secondary antibody (Jackson ImmunoResearch) was used at 1∶500. Images were obtained on a DeltaVision wide-field microscope and deconvolved. Fields were imaged in a random fashion and quantified by researchers blinded to treatment.

### Statistical analysis

Data were statistically analyzed using either ANOVA or Student's t-test using the GraphPad Prism software. Differences between treated samples and the control were considered significant at the level of 0.05.

## Results

### Autophagic killing of *M. tuberculosis* by alveolar macrophages

**Figure 1:**
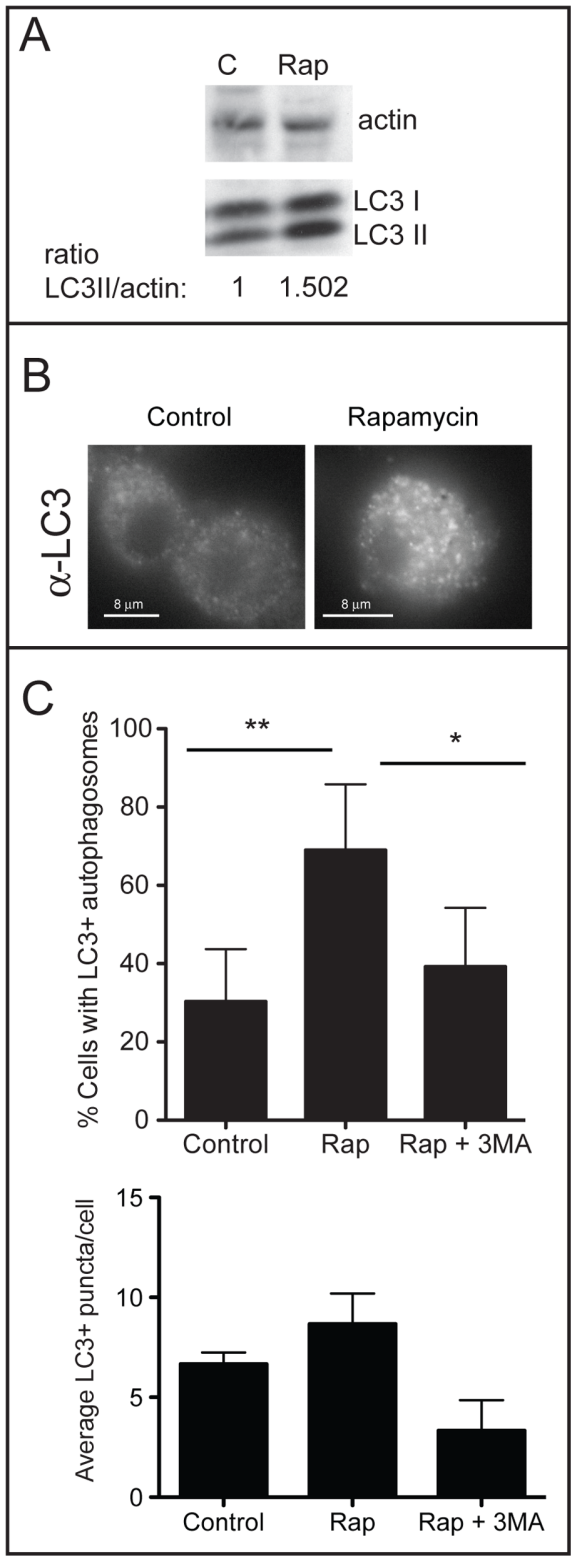
Assessment of autophagy in alveolar macrophages from adult RM. A. Western analysis was performed on cell lysates from resting control (C) or autophagic (Rap) alveolar macrophages using antibody against LC-3 and actin. The LC-3 antibody reacts with both free cytosolic LC3-I (18 kDa) and membrane associated LC3-II (16 kDa). Levels of LC3-II were normalized to actin by densitometry and the ratio of LC3-II/actin given below the blot. A representative image from a single rhesus macaque (RM) sample is shown. B. Immunofluorescence microscopy was performed on control and rapamycin-treated alveolar macrophages using primary antibody against LC-3. C. The number of cells possessing LC-3+ puncta (top) and the average number of LC3+ vacuoles in LC-3+ cells (bottom) were quantified (n = 6 NHP samples, n>50 macrophages in each condition). The difference between the number of cells possessing LC-3+ puncta was significantly different between control and autophagic cells (***, *p*<0.001; **, *p*<0.01; ANOVA).

Autophagy contributes to innate immunity by clearing cytoplasmic and intra-vacuolar pathogens, and we have previously demonstrated that the induction of autophagy in murine bone marrow-derived macrophages promotes clearance of *M. tuberculosis* [8], [24]. To assess autophagic clearance of *M. tuberculosis* by alveolar macrophages, we obtained macrophages from Rhesus macaques (RM) bronchial alveolar lavage (BAL) fluids. Autophagy was induced in RM alveolar macrophages using rapamycin. This pharmacological treatment was chosen over IFN-γ, a physiologically relevant inducer of autophagy, because rapamycin promotes delivery of the bacterium to the lysosome but does not promote the generation of ROI and RNI, which could complicate interpretation of our results. Phenotypes associated with the induction of autophagy were confirmed by Western blot analysis on total cell lysates using antibody against the autophagy marker LC3 (representative image, Fig. 1A). The LC3 antibody reacts with both free cytosolic LC3-I and autophagosomal membrane-associated LC3-II. We observed an increase in the relative LC3-II levels in rapamycin-treated cells, consistent with up-regulation of the autophagy pathway. We also examined resting and autophagic macrophages by immunofluorescence microscopy. There was an increase in the prevalence and number of LC3+ puncta in autophagic macrophages relative to control macrophages or autophagic macrophages treated with the phosphoinositide 3-kinase inhibitor 3-methyladenine (3-MA), a classic inhibitor of autophagy (Fig. 1B, C). These results are consistent with phenotypes described for murine and human macrophages or macrophage-like cell lines [25].

**Figure 2:**
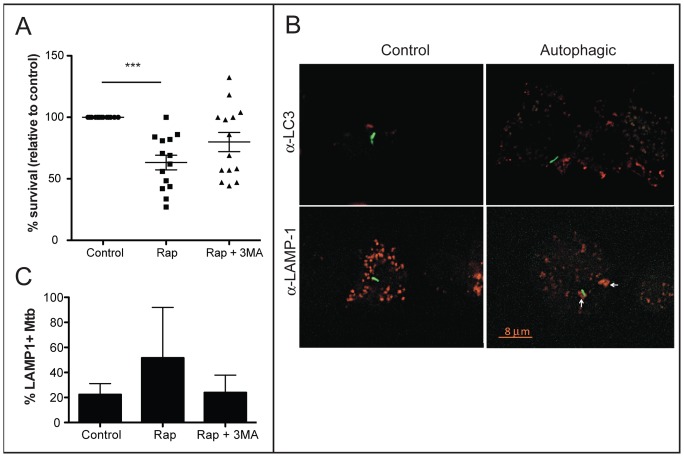
Bactericidal capacity of autophagic macrophages from adult RM. A. Alveolar macrophages were infected at an MOI of 5∶1 with *M. tuberculosis* CDC1551. Bacterial colony forming units (cfu) were determined following control treatment, 4 h treatment with 50 µg/mL rapamycin (rap) to induce autophagy, and 4 h treatment with 50 µg/mL rapamycin and 10 mM 3-methyladenine (3-MA) to block autophagy. Viability is expressed as % survival relative to the number of viable bacteria in untreated resting control macrophages. Each symbol represents the average of three triplicate infections for each condition using RM sample. The average and standard deviation of all samples are shown. The difference between bacterial survival in control and autophagic macrophages was significant (**, *p*<0.01; ANOVA). B. Immunofluorescence microscopy was performed on control and autophagic alveolar macrophages infected with fluorescently labeled *M. tuberculosis* CDC1551 (green). Primary antibodies against either the autophagosomal marker LC3 (top) or the lysosomal marker LAMP (bottom) and DyeLight 594-conjugated secondary antibody were used. Arrows indicate a representative mycobacterium co-localized with LAMP-1. C. The number of *M. tuberculosis* that co-localize with LAMP-1 were quantified. The difference between control and autophagic cells was not significantly different (p = 0.0586; ANOVA; n = 6 NHP samples).

To assess autophagic bactericidal activity, alveolar macrophages were infected at an MOI of 5∶1 with the *M. tuberculosis* clinical isolate CDC1551. Following infection, autophagy was induced using rapamycin. These experimental conditions did not impact macrophage viability or adherence (data not shown). To monitor bacterial survival in control and autophagic cells, infected macrophages were harvested, lysed, and serial dilutions plated to agar plates to enumerate mycobacterial colony forming units (cfu). Our results indicate that autophagic alveolar macrophages are significantly more bactericidal than untreated control macrophages (Fig. 2A). Similar bactericidal capacities of alveolar macrophages were observed upon infection of *M. tuberculosis* at a low MOI (1∶1) and high MOI (10∶1) (Fig S1A). Autophagic killing was prevented upon addition of 3-MA to block fusion of bacteria-containing vacuoles with the lysosome. Bafilomycin A, another inhibitor of autophagy, also effectively blocked autophagic killing (Fig. S2A). Autophagic delivery of fluorescently-labeled *M. tuberculosis* to the lysosome of infected alveolar macrophages was quantified following immunofluorescence microscopy (representative image, Fig. 2B). Following the induction of autophagy, there was a trend toward increased colocalization of *M. tuberculosis* with LAMP-1-positive vacuoles (Fig. 2C, *p* = 0.0568), consistent with the autophagic killing mechanism that we described previously for murine primary macrophages.

### Examination of potential age-related defects in RM alveolar macrophages

The elderly are more susceptible to respiratory infections including tuberculosis, and defects in innate immune responses may contribute to this increased vulnerability. Potential defects reported in the literature include, but are not limited to, phagocytic capacity, production of reactive nitrogen intermediates, chemotaxis, and signal transduction. Therefore, having established an *ex vivo* system for examining *M. tuberculosis* host-pathogen interactions, we utilized RM alveolar macrophages to determine if innate immune functions of alveolar macrophages were impaired in elderly hosts.

**Figure 3:**
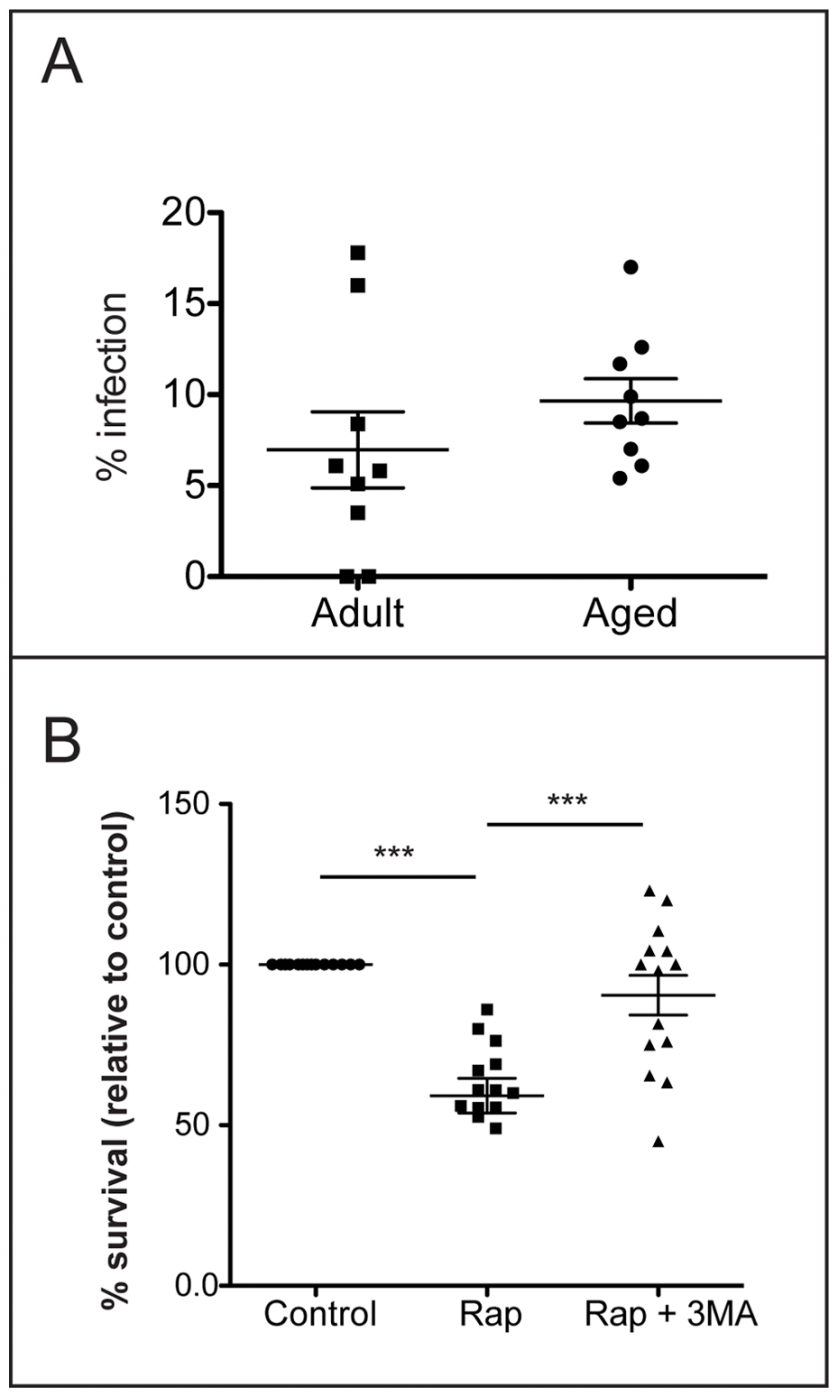
Comparison of innate immune functions of alveolar macrophages of adult and aged RM. A. Phagocytic capacity of alveolar macrophages from adult and aged RM was measured. Alveolar macrophages were incubated with fluorescently labeled *M. tuberculosis* CDC1551 for 45 minutes, and then extracellular bacteria removed by washing. Cells were fixed, and infected macrophages were observed by fluorescence microscopy. A macrophage was considered infected if it had internalized at least one bacterium. Each symbol represents the % infection of each RM sample. The average and standard deviation of all samples for each group is shown. n>100 macrophages for each RM sample. B. Alveolar macrophages from aged RM were infected at an MOI of 5∶1 with *M. tuberculosis* CDC1551. Bacterial cfu were determined following control treatment, 4 h treatment with 50 µg/mL rapamycin (rap) to induce autophagy, and 4 h treatment with 50 µg/mL rapamycin and 10 mM 3-methyladenine (3-MA) to block autophagy. Viability is expressed as % survival relative to the number of viable bacteria in untreated resting control macrophages. Each symbol represents the average of three triplicate infections for each condition using RM sample. The average and standard deviation of all samples are shown. The difference between bacterial survival in control and autophagic macrophages was significant (***, *p*<0.001; *, *p*<0.05; ANOVA).

As part of their role in immune surveillance, macrophages phagocytose microbes and deliver them to the lysosome for degradation. The effect of aging on this property is unclear since investigators have obtained different results using macrophages from different tissue sources or species [26]. To determine whether there was an age-related defect in the phagocytic capacity, we quantified uptake of fluorescently-labeled *M. tuberculosis* by alveolar macrophages from young and old RM (>18 years old). Using an MOI of 5∶1, roughly 8% of alveolar macrophages contained at least one bacterium (Fig. 3A). In general, we noted that primary alveolar macrophages are not highly phagocytic. When we used the same MOI to infect murine bone marrow-derived macrophages in a parallel experiment, 60% infection was achieved.

A growing literature reports that aging cells possess defects in autophagy [24]. To determine whether there was reduced autophagic clearance of *M. tuberculosis* in macrophages from old RM, we infected alveolar macrophages isolated from aged RM with *M. tuberculosis*. Autophagic alveolar macrophages from old RM were significantly more bactericidal than untreated control macrophages and this effect was reversed upon addition of 3-MA or Bafilomycin A (Fig. 3B, S2B). Similar bactericidal capacity of alveolar macrophages from old RM was observed upon infection of *M. tuberculosis* at a low MOI (1∶1) and high MOI (10∶1) (Fig S1B). There was no significant difference in the ability of autophagic alveolar macrophages from young and old RM to control *M. tuberculosis* infection (Fig. 2A, 3B).

Autophagy is a constitutive process in eukaryotic cells [27]. To determine whether there were differences in the baseline or intrinsic autophagic bactericidal capacity of primary alveolar macrophages, we performed *M. tuberculosis* infections in the presence of 3-MA or bafilomycin A. The difference between control and 3-MA- or Bafilomycin A-treated macrophages in control of *M. tuberculosis* infection was not significant (Fig. S2). We noted an increase in the bactericidal capacity of alveolar macrophages from some, but not all, adult RM upon inhibition of steady-state autophagy. This range of results likely reflects intrinsic differences between the immune homeostasis of individual RM. Further, our analysis did not reveal significant age-related differences in the constitutive levels of autophagy in alveolar macrophages measured as *M. tuberculosis* survival. Overall, these experiments demonstrate that inhibition of constitutive autophagy has a minimal effect on *M. tuberculosis* viability. These results may reflect the ability of *M. tuberculosis* to actively inhibit autophagy in a manner that depends on Rv2416c, known as the “enhanced intracellular survival” protein Eis [28].

**Figure 4:**
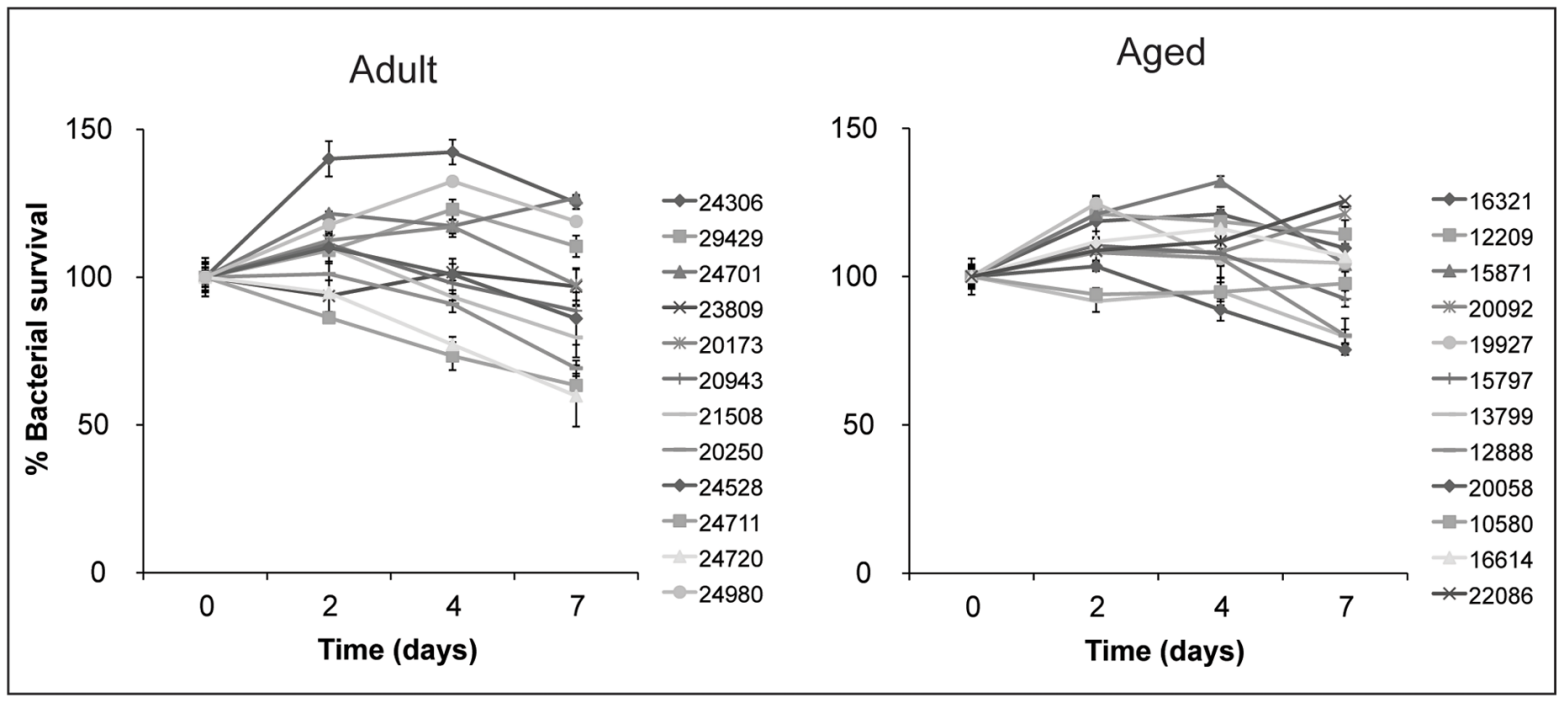
Bactericidal capacity of resting alveolar macrophages. Alveolar macrophages were infected at an MOI of 5∶1 with *M. tuberculosis* CDC1551. Bacterial survival was followed over seven days by harvesting the infected monolayers at the indicated timepoints and plating serial dilutions. The assay was performed in triplicate and the average and standard deviation for each time point are shown for each RM sample. The reference number for each RM is noted in the legend. Bacterial survival in alveolar macrophages from adult (left) and aged (right) RM is shown. There was no significant difference in mycobacterial survival between groups at t = 7d (Student's t-test, *p* = 0.0693).

Since our initial studies did not reveal differences in autophagic clearance of *M. tuberculosis* by alveolar macrophages from young and old RM, we asked if there were differences in the ability of these two macrophage groups to control *M. tuberculosis* infection over a longer time course. Alveolar macrophages were infected with *M. tuberculosis* and the infection followed for seven days. Infected macrophages remained healthy over the course of the infection and we did not observe any signs of detachment or cytotoxicity (data not shown). Alveolar macrophages from young and old RM controlled infection in a comparable fashion (Fig. 4). While our sample size is limited, these data suggest that resting macrophages from young and aged animals have the same capacity to control *M. tuberculosis* infection.

## Discussion

Susceptibility to *M. tuberculosis* is complex and multifactorial and includes genetic susceptibility, immune status, and age of the host. The elderly are more susceptible to respiratory infections including tuberculosis, and the incidence of tuberculosis in elderly white Americans has increased from 29% to 58% within the last 10 years (American Lung Association). These numbers reflect both reactivation of latent infections and primary infections that are increasingly common in long-term care facilities. Therefore the impact of an aging immune system on *M. tuberculosis* infection is increasingly relevant. Upon inhalation and infection of the lung, *M. tuberculosis* initially encounters and infects alveolar macrophages. Studies in animal models and human monocyte-derived macrophages indicate aging affects macrophage function; although interpretation of these data is complicated by differences in species, cell source, and experimental design. While previous work demonstrated that alveolar macrophages from old mice had reduced uptake of latex beads [29], no age-related defects were noted in phagocytosis of bacteria by rat alveolar macrophages [30]. Our study investigated the impact of age on phagocytosis and autophagy-mediated killing functions of alveolar macrophages isolated from macaques, which are increasingly used to investigate host-pathogen interactions during *M. tuberculosis* infection as well as the impact of age on immune function.

Autophagy is a highly conserved pathway that is involved in many cellular processes including protein and organelle quality control and pathogen clearance. Defects in or reduced autophagy is implicated in aging and the onset of age-related pathologies including neurodegenerative diseases such as Alzheimers, Parkinsons and Huntingtons [24]. It is proposed that reduced autophagosome formation and fusion with the lysosome in the context of infectious disease could contribute to immunosenescence [22]. However, our data indicate that alveolar macrophages from aged RM are as competent as those from young RM to control bacterial replication and kill *M. tuberculosis* upon the induction of autophagy. In addition, our data did not reveal any significant differences in phagocytic capacity between alveolar macrophages from young and old rhesus macaques. These results suggest species or cell type differences in autophagy should be considered when examining interactions between human pathogens and the innate or adaptive immune system.

We also characterized the ability of alveolar macrophages from young and old RM to control *M. tuberculosis* replication over an extended time course. Our data do not indicate significant differences between control of *M. tuberculosis* infection by macrophage from young and old animals. Rhoades and Orme previously examined the effect of age on macrophage response to *M. tuberculosis* infection using bone marrow-derived macrophages generated from young and old mice. This study also found no significant differences in the ability of macrophages to control *M. tuberculosis* survival or replication [31]. While we have focused on the impact of aging on the ability of alveolar macrophages to contain *M. tuberculosis* infection, a number of other aspects of macrophage function could be impaired such as cytokine production or antigen presentation. Murine models clearly indicate that TLR expression and cytokine release by macrophages declines with age [32]–[34], while the results obtained from human peripheral monocytes vary [35]. Cytokine production by IFN-γ activated alveolar macrophages from old mice was lower compared with young mice [29]. However, it should be noted that work by Orme and colleagues did not identify any defects in cytokine or MHC II expression *in vitro* following *M. tuberculosis* infection of bone marrow derived macrophages isolated from aged mice [31]. Moreover, cytokine response by alveolar macrophages was not reduced in aged murine alveolar macrophages following low-dose *M. tuberculosis* infection [36]. Therefore our data from RM, in combination with data obtained using the aged mouse model, seem to exclude macrophage dysfunction in early timepoints following infection.

Instead, increased susceptibility to *M. tuberculosis* in aged hosts likely results from alterations in the T cell repertoire and response. Initial studies demonstrated increased susceptibility of old mice to i.v. infection with *M. tuberculosis* that was attributed to poor CD4+ T cell responses [37], [38]. Despite the failure of old mice to control *M. tuberculosis* infection, subsequent work demonstrated that upon low-dose infection, old mice possessed a transient early resistance to *M. tuberculosis* that was mediated by CD8+ T cells [39], [40]. Turner has therefore proposed a model whereby old mice are able to mount an early innate response, but delayed generation of an adaptive immune response results in higher bacterial burden and inflammation [41]. Further investigation into the mechanisms of effective immune control of *M. tuberculosis* and application of this knowledge to the benefit of the elderly population is necessary. The use of animal models, in particular NHP, that recapitulate phenotypes associated with human immunosenescence and latent tuberculosis infection and reactivation will likely provide novel insights into this public health problem.

## Supporting Information

Figure S1 **Bactericidal capacity of autophagic macrophages from RM at low and high MOI.** Alveolar macrophages from adult RM (A) or aged RM (B) were infected at an MOI of 1∶1 or 10∶1 with *M. tuberculosis* CDC1551. Bacterial colony forming units (cfu) were determined following control treatment, 4 h treatment with 50 µg/mL rapamycin (rap) to induce autophagy, and 4 h treatment with 50 µg/mL rapamycin and 10 mM 3-methyladenine (3-MA) to block autophagy. Viability is expressed as % survival relative to the number of viable bacteria in untreated resting control macrophages. Each symbol represents the average of three triplicate infections for each condition using RM sample. The average and standard deviation of all samples are shown. The difference between bacterial survival in control and autophagic macrophages was significant (*, *p*<0.05**; *p*<0.01: ***; *p*<0.001; ANOVA).
(TIF)

Figure S2 **Bactericidal capacity of autophagic macrophages from RM is blocked with bafilomycin A.** Alveolar macrophages from adult RM (A) or aged RM (B) were infected at an MOI of 5∶1 with *M. tuberculosis* CDC1551. Bacterial colony forming units (cfu) were determined following control treatment, 4 h treatment with 50 µg/mL rapamycin (rap) to induce autophagy, and 4 h treatment with 50 µg/mL rapamycin and 0.1 µM bafilomycin (baf) to block autophagy. Intrinsic levels of autophagy were block by treatment with 10 mM 3MA or 0.1 µM bafilomycin (baf). Viability is expressed as % survival relative to the number of viable bacteria in untreated resting control macrophages. Each symbol represents the average of three triplicate infections for each condition using RM sample. The average and standard deviation of all samples are shown. The difference between bacterial survival in control and autophagic macrophages was significant (*, *p*<0.05; ANOVA).
(TIF)

Table S1 **Age and sex of non-human primates.**
(DOCX)
