# Supplementary figures and images for: Autophagic Killing Effects against Mycobacterium tuberculosis by Alveolar Macrophages from Young and Aged Rhesus Macaques
Source: PLoS One. 2013 Jun 18;8(6):e66985. doi: 10.1371/journal.pone.0066985 (PMC3688994; doi:10.1371/journal.pone.0066985)

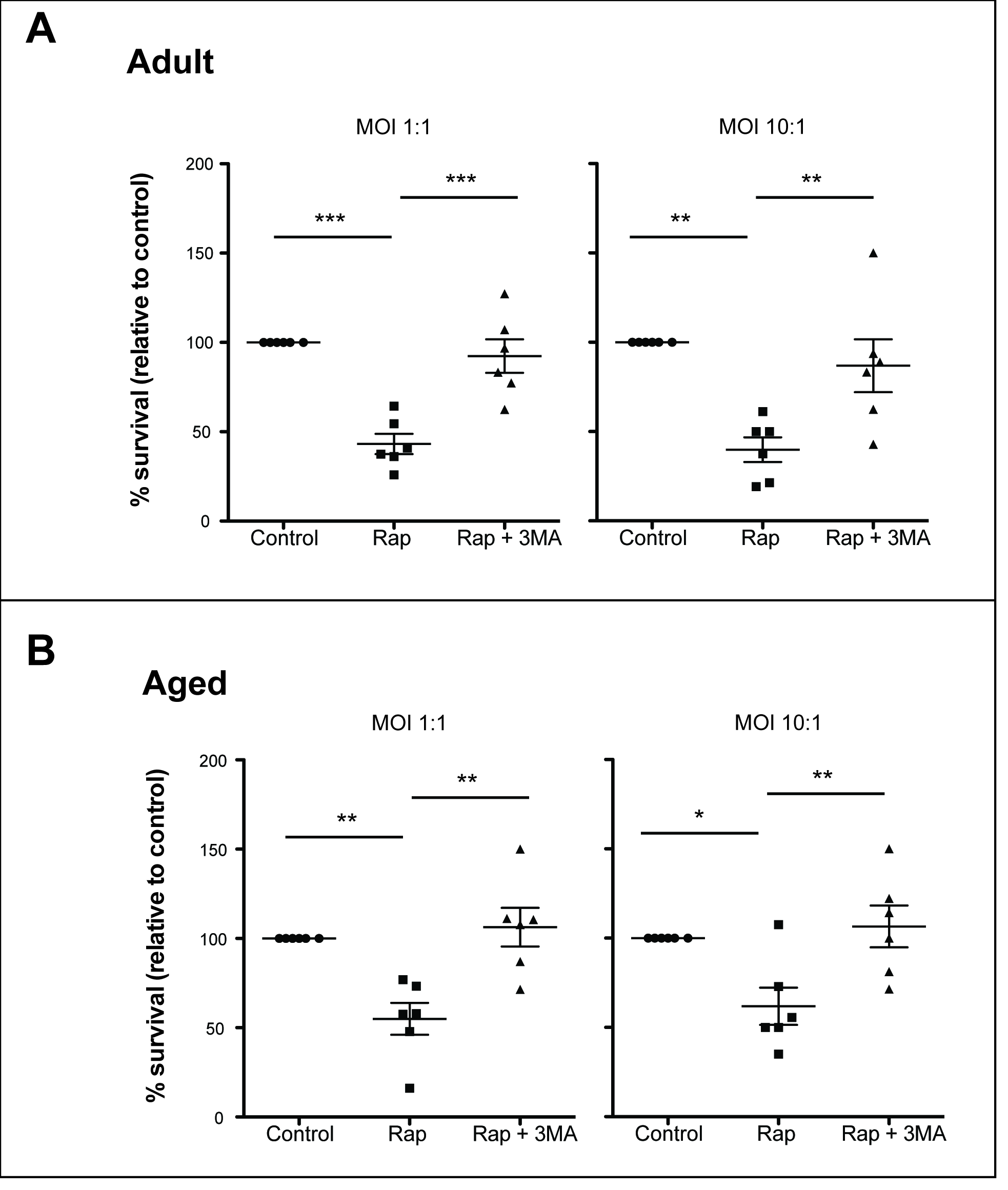

Supplement: Figure S1 — Bactericidal capacity of autophagic macrophages from RM at low and high MOI. Alveolar macrophages from adult RM (A) or aged RM (B) were infected at an MOI of 1∶1 or 10∶1 with M. tuberculosis CDC1551. Bacterial colony forming units (cfu) were determined following control treatment, 4 h treatment with 50 µg/mL rapamycin (rap) to induce autophagy, and 4 h treatment with 50 µg/mL rapamycin and 10 mM 3-methyladenine (3-MA) to block autophagy. Viability is expressed as % survival relative to the number of viable bacteria in untreated resting control macrophages. Each symbol represents the average of three triplicate infections for each condition using RM sample. The average and standard deviation of all samples are shown. The difference between bacterial survival in control and autophagic macrophages was significant (*, p<0.05**; p<0.01: ***; p<0.001; ANOVA). (TIF) [file pone.0066985.s001.tif]

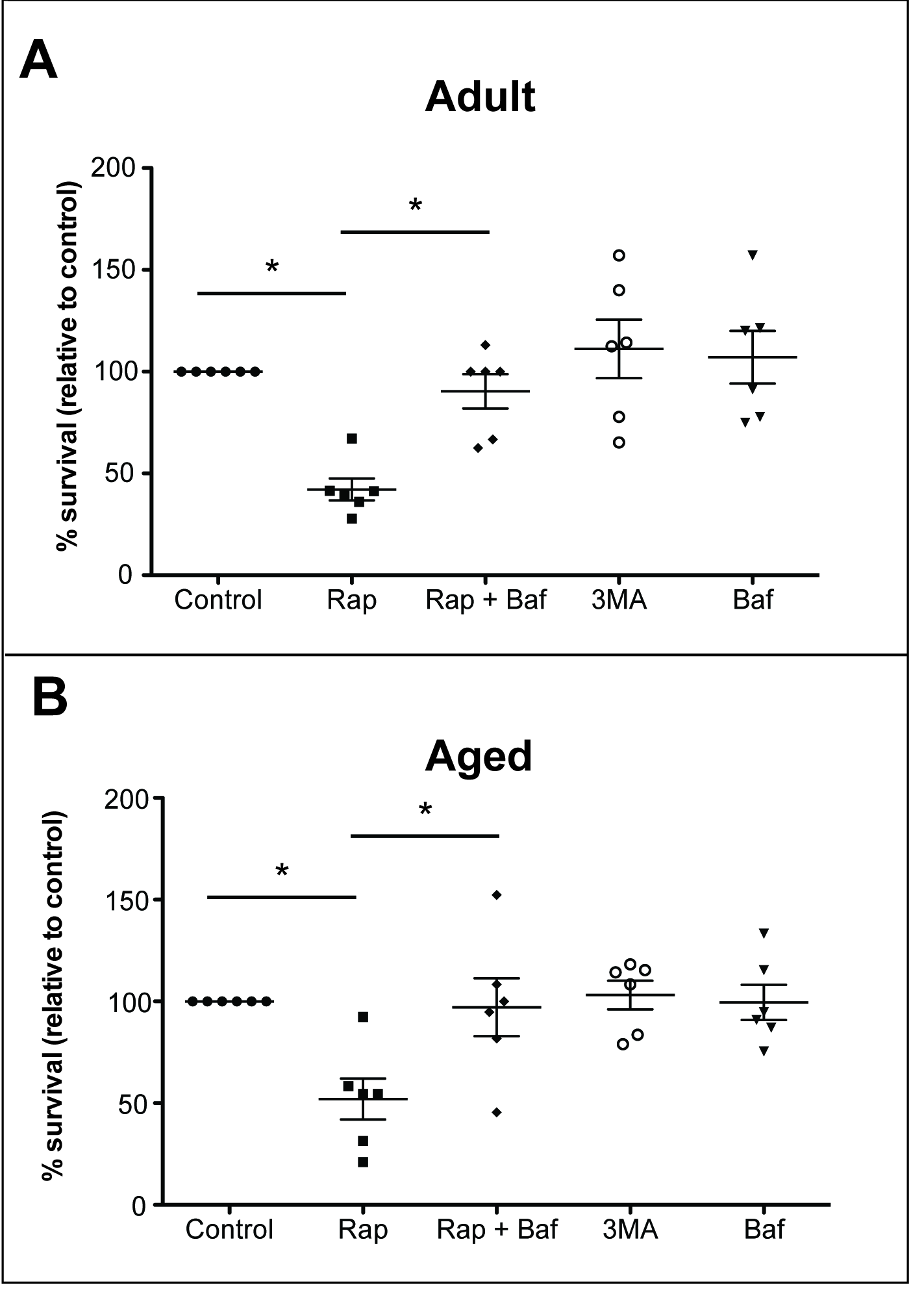

Supplement: Figure S2 — Bactericidal capacity of autophagic macrophages from RM is blocked with bafilomycin A. Alveolar macrophages from adult RM (A) or aged RM (B) were infected at an MOI of 5∶1 with M. tuberculosis CDC1551. Bacterial colony forming units (cfu) were determined following control treatment, 4 h treatment with 50 µg/mL rapamycin (rap) to induce autophagy, and 4 h treatment with 50 µg/mL rapamycin and 0.1 µM bafilomycin (baf) to block autophagy. Intrinsic levels of autophagy were block by treatment with 10 mM 3MA or 0.1 µM bafilomycin (baf). Viability is expressed as % survival relative to the number of viable bacteria in untreated resting control macrophages. Each symbol represents the average of three triplicate infections for each condition using RM sample. The average and standard deviation of all samples are shown. The difference between bacterial survival in control and autophagic macrophages was significant (*, p<0.05; ANOVA). (TIF) [file pone.0066985.s002.tif]
