# Supplementary material for: Autophagic Killing Effects against Mycobacterium tuberculosis by Alveolar Macrophages from Young and Aged Rhesus Macaques
Source: PLoS One. 2013 Jun 18;8(6):e66985. doi: 10.1371/journal.pone.0066985 (PMC3688994; doi:10.1371/journal.pone.0066985)
Supplement: Table S1 — Age and sex of non-human primates. (DOCX) [file pone.0066985.s003.docx]

**Table S1. Age and sex of non-human primates**

| **ID** | **Age** | **Sex** |
| --- | --- | --- |
| 24306 | 3 | F |
| 29429 | 7 | F |
| 24701 | 3 | F |
| 23809 | 4 | M |
| 20173 | 9 | M |
| 20943 | 7 | F |
| 21508 | 7 | M |
| 20250 | 9 | M |
| 24528 | 4 | M |
| 24711 | 3 | F |
| 24720 | 5 | F |
| 24980 | 2 | F |
| 16321 | 17 | F |
| 12209 | 27 | M |
| 15871 | 18 | F |
| 22086 | 24 | F |
| 20092 | 25 | F |
| 19927 | 20 | F |
| 15797 | 20 | F |
| 13799 | 22 | F |
| 12888 | 26 | M |
| 20058 | 26 | F |
| 10580 | 30 | F |
| 16614 | 16 | F |
